# Supplementary material for: Evaluating the practical aspects and performance of commercial single-cell RNA sequencing technologies
Source: NAR Genom Bioinform. 2026 Jan 6;8(1):lqaf215. doi: 10.1093/nargab/lqaf215 (PMC12770963; doi:10.1093/nargab/lqaf215)
Supplement: lqaf215_Supplemental_Files [file lqaf215_supplemental_files.zip › 20251203_supplemental_figures.pdf]

Supplemental figure 1

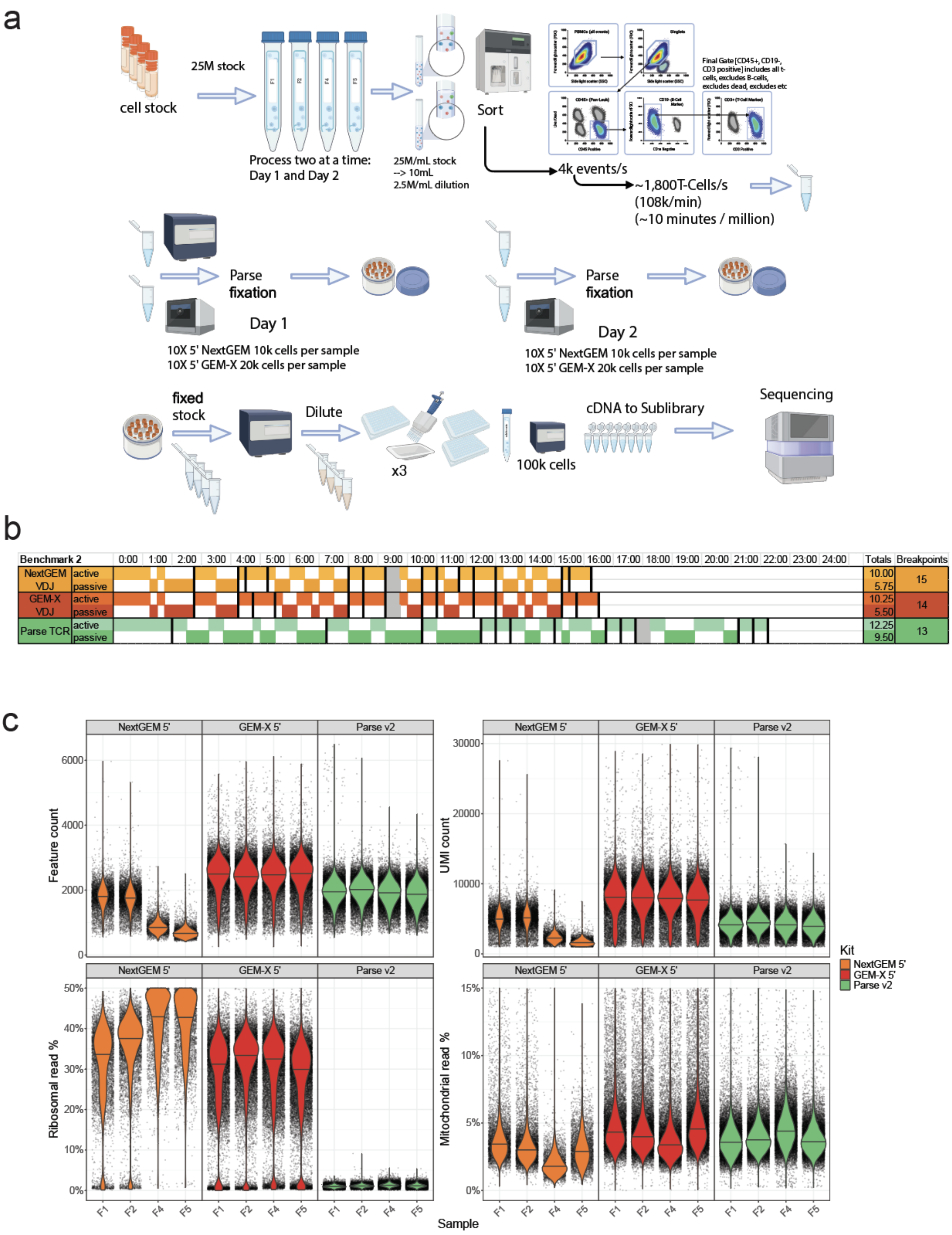

Supplementary Figure 1: Benchmark 2 experimental workflow, timing, and QC. Created in BioRender. Elz, A. (2025) https://BioRender.com/bfgq52z

Workflow diagram of different steps required for each platform used in the Benchmark 2 experiment from the same cell suspension from sorting T cells to library sequencing. Two samples were processed at a time on two separate days for each platform.

a. Summary of time taken to execute each kit from sample preparation to library completion in terms of hours spent hands-on (active) and time spent waiting (passive). Stopping points are indicated with redlines and the number of stopping points is summarized in the rightmost column. Gray indicates an overnight stopping point.
b. Cell-level QC metric distributions for 5' assays. Each point is a single cell. The violin overlays show the density distribution of cells in the sample with the median indicated by the line. From the top left going clockwise, the metrics are: feature (gene) count, UMI (transcript) count, % of transcripts from mitochondrial genes, % of transcripts from ribosomal genes.

Supplemental figure 2

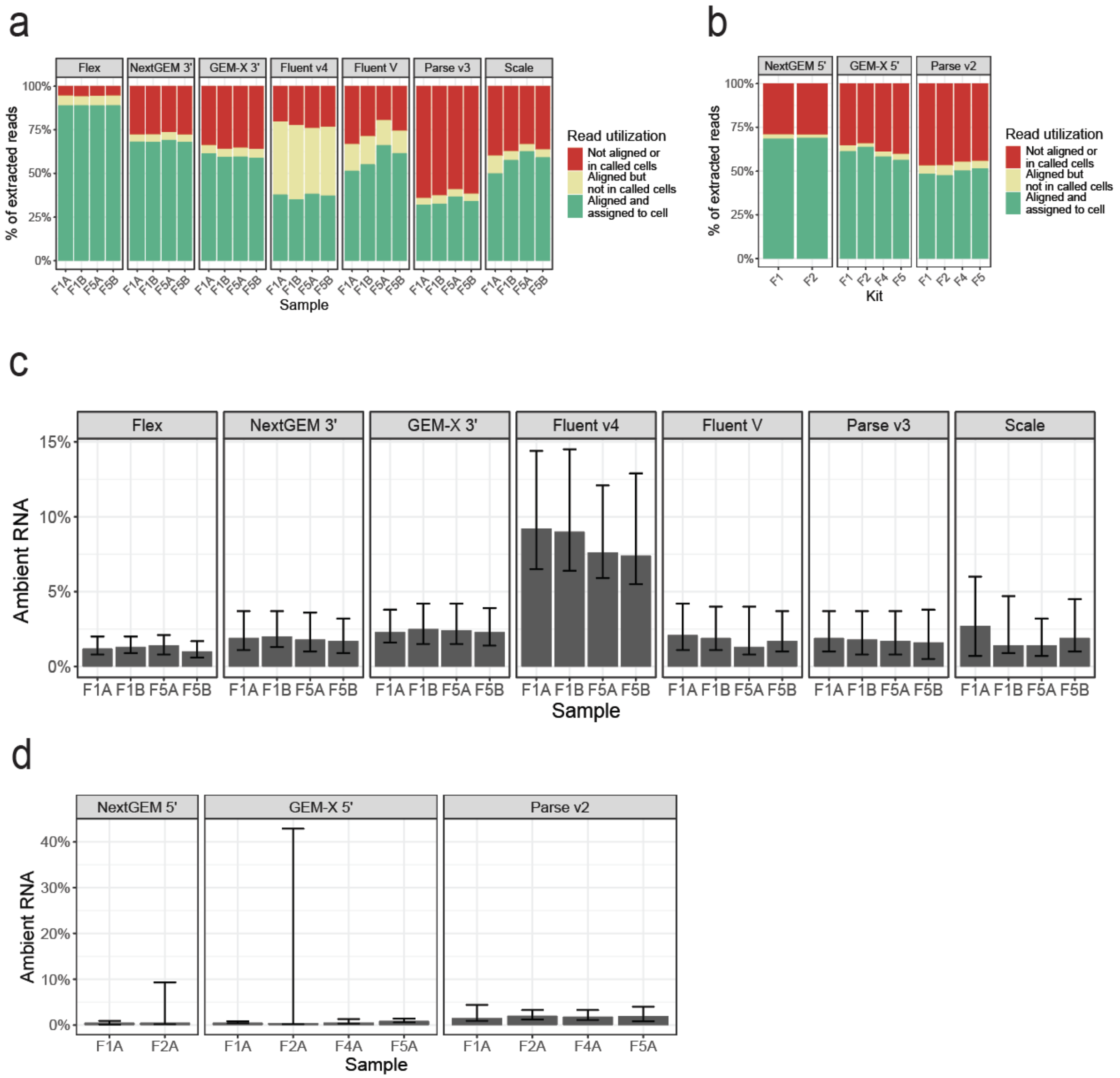

Supplementary Figure 2: Ambient RNA and read utilization

a-b. Percentage of demultiplexed reads from Benchmark 1 (a) and Benchmark 2 (b) WT gene expression libraries that contributed to the final counts matrix for each pipeline. Reads had to align to reference genome features and be from valid cells to contribute to the counts matrix. Aligned reads not in called cells could be included in the counts matrix with different cell calling criteria.

c-d. Estimated percentage of reads from ambient (i.e. extra-cellular) RNA. Error bars indicate 95% confidence interval for ambient contamination estimates. Ambient RNA was estimated but not removed from the data.

Supplemental figure 3

a

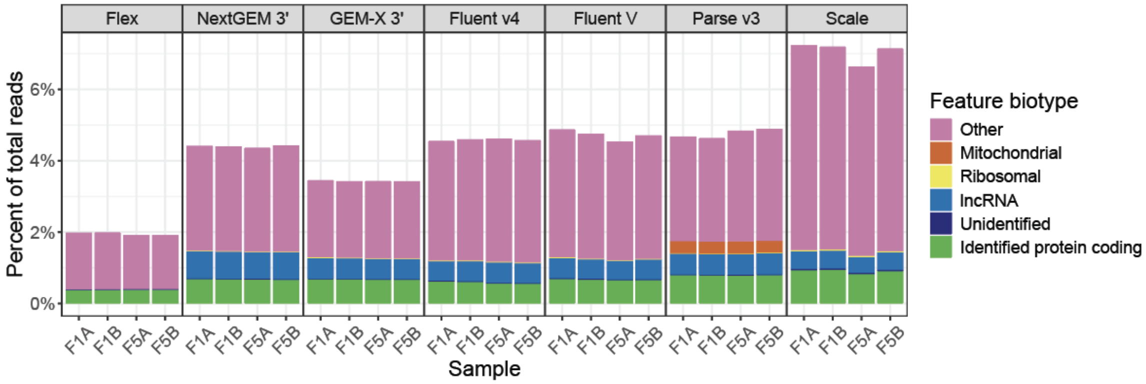

b

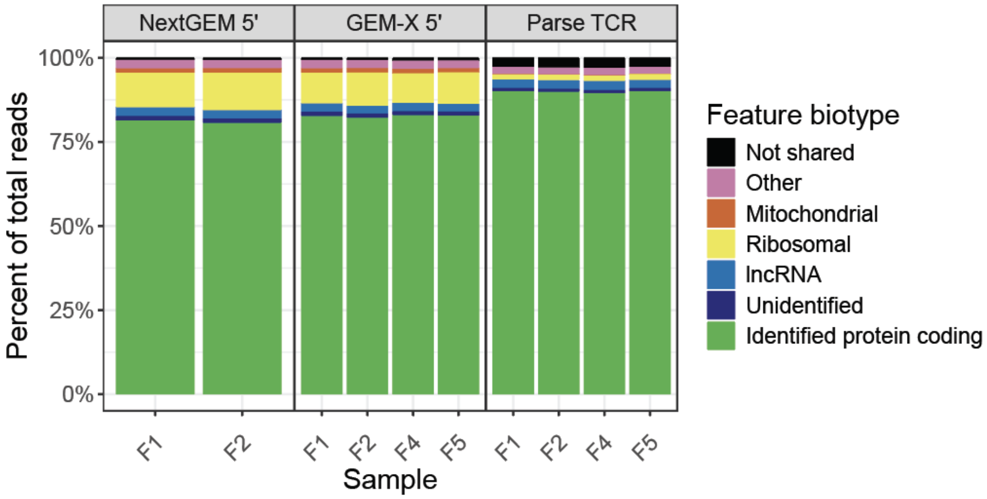

c

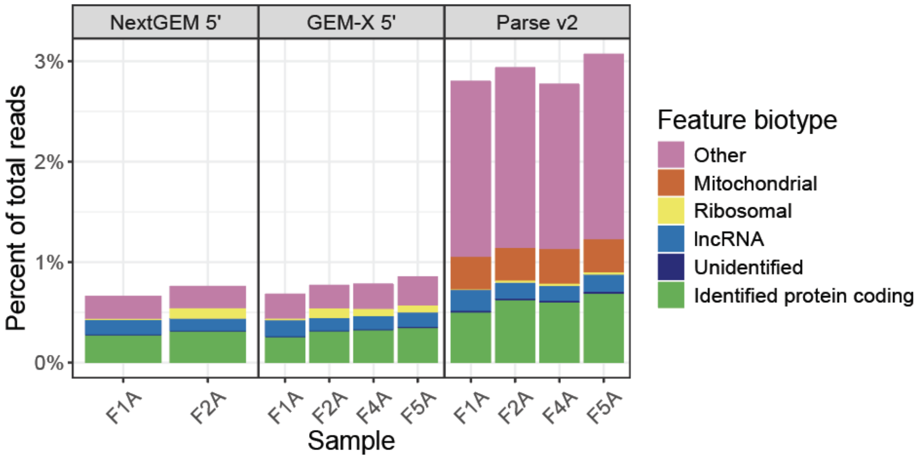

Supplementary Figure 3: Gene recovery

a. Read mapping feature classifications for the non-shared features from 3’ kits. These features were not identified across all kits but may have been found in more than one kit. Identified protein-coding features have gene symbols. Unidentified features have location information or unique identifiers, but no gene symbol. “Other” indicates a feature could not be binned as only one of the other biotypes.
b. Read mapping feature classification for all 5’ data. See classification explanations in (a).
c. Read mapping feature classifications for the non-shared features from 5’ kits. See classification explanations in (a).

Supplemental figure 4a

Flex

		Seurat						SingleR					
		pbmcsca			pbmc3k			Mona			HPCA		
Cluster	Cell count	Label	Score	% called	Label	Score	% called	Label	Score	% called	Label	Score	% called
0	2602	CD4+ T	0.99	100.00	CD4+ T	0.98	100.00	CD4+ T	0.29	97.89	T	0.75	99.96
1	2028	Classical monocyte	1.00	99.16	Classical monocyte	0.99	96.65	Monocyte	0.07	87.28	Monocyte	0.07	90.38
2	2017	NK	0.91	96.93	NK	0.98	99.26	CD8+ T	0.10	69.96	NK	0.48	99.11
3	2002	CD8+ T	0.84	97.05	CD8+ T	0.77	92.76	CD8+ T	0.10	71.93	T	0.15	89.46
4	1981	B	1.00	99.04	B	1.00	98.94	B	0.09	93.99	B	0.07	97.32
5	1973	CD4+ T	0.96	100.00	CD4+ T	1.00	100.00	CD4+ T	0.25	94.98	T	0.69	99.44
6	1920	CD8+ T	0.73	74.01	CD8+ T	0.69	59.17	CD8+ T	0.10	98.02	NK	0.12	49.64
7	1872	CD4+ T	0.91	94.07	CD4+ T	0.97	95.19	CD4+ T	0.19	92.41	T	0.56	97.65
8	1681	Classical monocyte	1.00	99.82	Classical monocyte	0.98	99.52	Monocyte	0.07	93.16	Monocyte	0.08	93.63
9	1649	CD4+ T	1.00	100.00	CD4+ T	0.96	100.00	CD4+ T	0.25	96.06	T	0.70	99.76
10	1634	B	1.00	100.00	B	1.00	99.82	B	0.10	96.88	B	0.07	98.10
11	1576	CD4+ T	0.81	83.38	CD4+ T	0.96	91.88	CD8+ T	0.00	97.02	T	0.50	96.83
12	1519	CD4+ T	1.00	100.00	CD4+ T	0.91	100.00	CD4+ T	0.23	94.54	T	0.66	99.01
13	1446	CD4+ T	1.00	99.86	CD4+ T	0.94	100.00	CD8+ T	0.06	97.79	T	0.73	99.17
14	1391	CD8+ T	0.77	83.68	CD8+ T	0.83	91.59	CD8+ T	0.00	51.62	T	0.24	94.25
15	1254	Non–classical monocyte	0.97	96.97	Non–classical monocyte	1.00	98.56	Monocyte	0.08	85.09	Monocyte	0.08	94.42
16	1242	CD8+ T	0.75	84.30	CD8+ T	0.80	79.23	CD8+ T	0.10	90.02	T	0.20	89.86
17	1067	CD4+ T	1.00	100.00	CD4+ T	0.95	100.00	CD8+ T	0.05	99.44	T	0.69	99.91
18	998	CD4+ T	0.99	100.00	CD4+ T	0.84	100.00	CD4+ T	0.22	91.18	T	0.68	98.90
19	879	CD4+ T	0.93	99.54	CD4+ T	0.99	100.00	CD4+ T	0.19	92.72	T	0.38	97.84
20	805	CD4+ T	0.95	100.00	CD4+ T	1.00	100.00	CD4+ T	0.23	94.41	T	0.65	98.88
21	781	Classical monocyte	0.99	61.08	Classical monocyte	0.92	52.50	Monocyte	0.07	52.75	Monocyte	0.08	94.49
22	658	NK	0.71	74.16	NK	0.84	78.12	CD8+ T	0.10	62.16	NK	0.32	95.14
23	285	Megakaryocyte	1.00	74.39	Megakaryocyte	0.95	74.39	Lymphocyte progenitor	0.07	41.40	Megakaryocyte	0.11	65.96
24	144	pDC	0.54	77.78	Dendritic	0.90	100.00	Dendritic	0.15	99.31	Myeloid	0.00	57.64
25	114	CD4+ T	0.98	88.60	CD4+ T	0.99	91.23	CD4+ T	0.25	73.68	T	0.64	97.37
26	96	CD4+ T	0.73	40.62	CD4+ T	0.62	52.08	CD4+ T	0.06	20.83	T	0.12	30.21
27	61	CD4+ T	0.60	57.38	CD4+ T	0.62	95.08	Granulocyte	0.08	45.90	Granulocyte	0.19	32.79
28	35	Classical monocyte	0.89	94.29	Classical monocyte	0.66	42.86	Monocyte	0.26	68.57	Monocyte	0.11	88.57
29	29	CD8+ T	0.65	51.72	CD4+ T	0.26	62.07	CD8+ T	0.10	79.31	NK	0.62	62.07

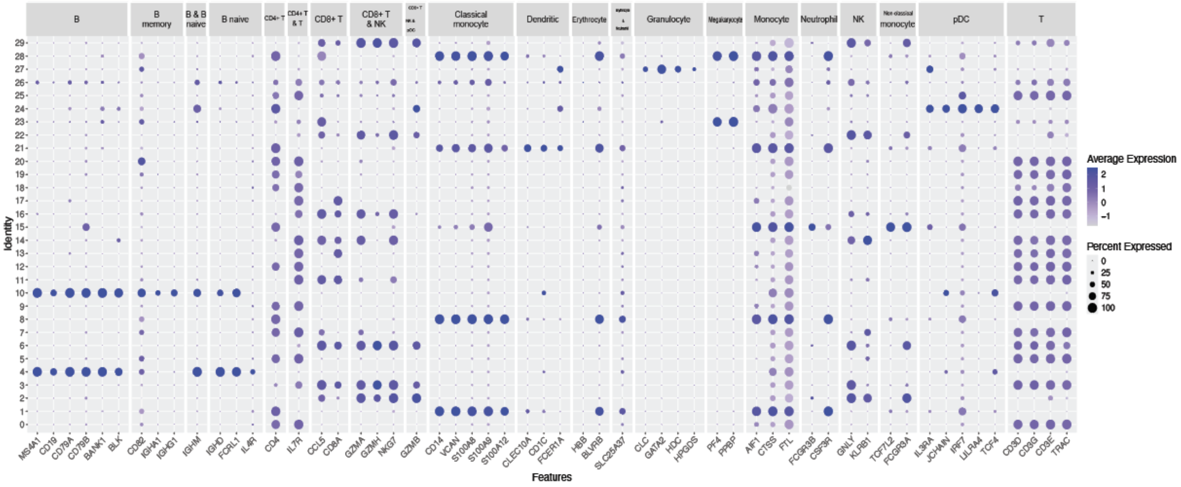

Leiden clustering

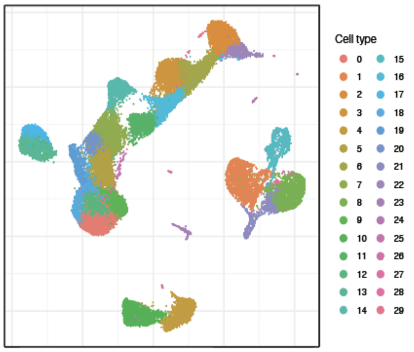

Final annotations

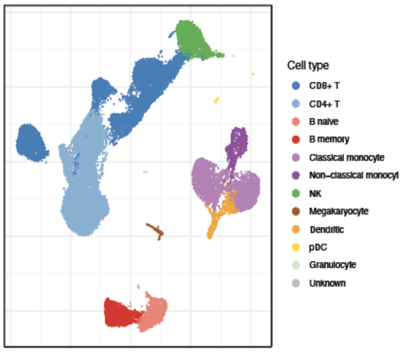

Cell label	Count	Proportion
CD4+ T	12411	0.35
CD8+ T	10673	0.30
Classical monocyte	3744	0.10
NK	2675	0.07
B naive	1981	0.06
B memory	1634	0.05
Non–classical monocyte	1254	0.04
Dendritic	781	0.02
Megakaryocyte	285	0.01
pDC	144	0.00
Unknown	96	0.00
Granulocyte	61	0.00

Supplemental figure 4b

NextGEM 3′

		Seurat						SingleR					
		pbmcsca			pbmc3k			Mona			HPCA		
Cluster	Cell count	Label	Score	% called	Label	Score	% called	Label	Score	% called	Label	Score	% called
0	4010	CD4+ T	1.00	99.83	CD4+ T	0.70	100.00	CD4+ T	0.17	83.42	T	0.21	98.33
1	3370	CD4+ T	1.00	99.97	CD4+ T	0.93	100.00	CD4+ T	0.13	72.64	T	0.16	96.14
2	2958	NK	0.79	71.60	NK	0.97	89.28	NK	0.00	72.24	NK	0.21	94.56
3	2817	CD4+ T	1.00	99.89	CD4+ T	0.86	100.00	CD4+ T	0.14	75.97	T	0.18	97.62
4	2791	CD8+ T	0.87	97.85	CD8+ T	0.92	96.74	T	0.08	34.65	T	0.10	73.84
5	2580	Classical monocyte	1.00	99.65	Classical monocyte	0.99	99.65	Monocyte	0.06	77.91	Monocyte	0.02	59.77
6	2547	B	1.00	99.92	B	1.00	99.92	B	0.08	80.64	B	0.07	90.58
7	2492	CD8+ T	0.81	87.04	CD8+ T	0.85	85.51	NK	0.00	41.97	T	0.09	51.93
8	2405	CD4+ T	0.97	99.42	CD4+ T	0.83	100.00	CD4+ T	0.16	82.62	T	0.19	97.21
9	2382	CD4+ T	0.91	96.73	CD4+ T	0.86	95.68	CD4+ T	0.10	64.69	T	0.15	94.21
10	2258	B	1.00	99.91	B	1.00	99.91	B	0.09	86.05	B	0.07	90.57
11	2239	CD4+ T	0.78	64.63	CD4+ T	0.81	66.95	T	0.08	41.76	T	0.14	92.27
12	1908	CD4+ T	1.00	99.84	CD4+ T	0.80	100.00	CD8+ T	0.07	55.66	T	0.16	97.12
13	1882	Classical monocyte	1.00	95.86	Classical monocyte	0.95	90.91	Monocyte	0.06	76.46	Monocyte	0.01	72.64
14	1625	Classical monocyte	1.00	99.94	Classical monocyte	1.00	99.75	Monocyte	0.00	70.09	Monocyte	0.02	45.91
15	1452	CD4+ T	1.00	100.00	CD4+ T	0.89	100.00	CD8+ T	0.07	56.13	T	0.15	96.76
16	1435	CD8+ T	0.70	56.79	CD8+ T	0.89	92.61	T	0.08	34.77	T	0.13	89.41
17	1398	Non–classical monocyte	0.88	94.92	Non–classical monocyte	1.00	94.71	Monocyte	0.06	80.26	Monocyte	0.00	65.67
18	1228	CD4+ T	0.96	98.29	CD4+ T	0.91	100.00	CD4+ T	0.10	67.67	T	0.14	94.87
19	1075	CD4+ T	0.91	94.98	CD4+ T	0.92	94.14	CD4+ T	0.10	66.70	T	0.16	94.88
20	983	CD4+ T	0.96	99.29	CD4+ T	0.96	99.69	CD4+ T	0.13	76.91	T	0.19	95.93
21	971	CD4+ T	0.89	93.92	CD4+ T	0.87	99.79	CD4+ T	0.11	68.59	T	0.12	88.36
22	618	Dendritic	0.72	78.16	Dendritic	0.95	91.42	Dendritic	0.07	49.19	Monocyte	0.05	59.55
23	474	Megakaryocyte	1.00	47.89	Megakaryocyte	0.94	48.73	B	0.05	39.24	T	0.12	50.84
24	395	CD8+ T	0.73	77.72	CD8+ T	0.89	93.92	NK	0.00	58.73	T	0.11	77.97
25	192	CD4+ T	0.51	38.54	NK	0.81	58.33	NK	0.00	50.00	NK	0.19	77.60
26	156	pDC	0.59	92.95	Dendritic	0.78	96.15	Dendritic	0.11	96.15	Myeloid	0.00	35.26
27	147	CD4+ T	0.92	95.92	CD4+ T	0.77	92.52	T	0.08	31.97	T	0.13	87.76
28	102	Classical monocyte	0.91	87.25	Classical monocyte	0.77	67.65	Monocyte	0.07	50.98	Monocyte	0.06	60.78
29	43	Classical monocyte	0.46	100.00	CD4+ T	0.59	100.00	B	0.00	83.72	BM	0.01	60.47

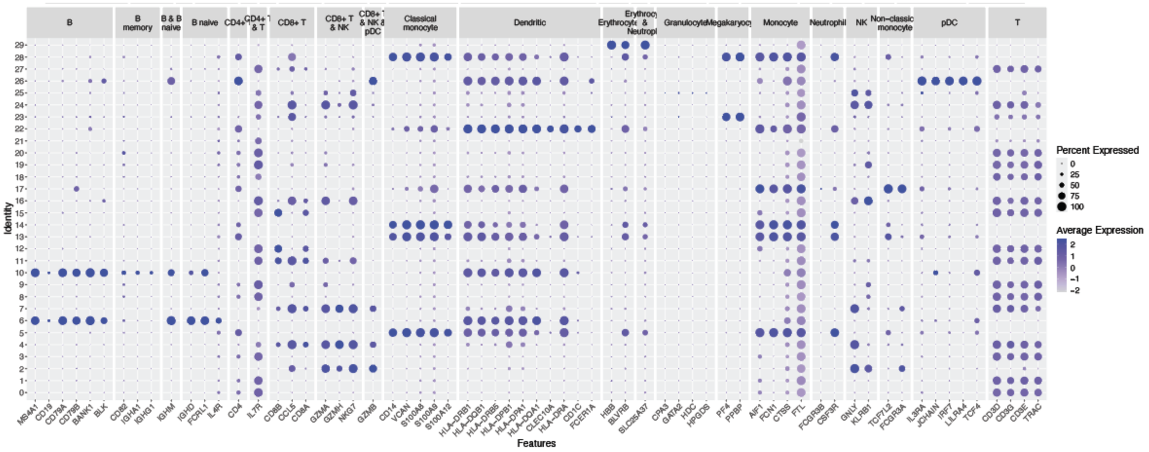

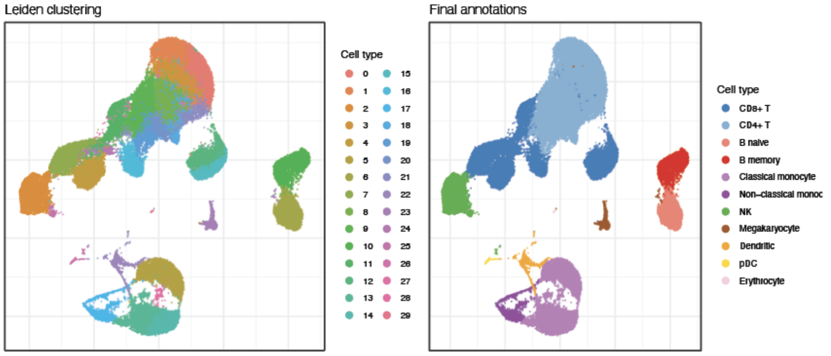

Cell label	Count	Proportion
CD4+ T	19241	0.39
CD8+ T	12859	0.26
Classical monocyte	6189	0.13
NK	3150	0.06
B naive	2547	0.05
B memory	2258	0.05
Non–classical monocyte	1398	0.03
Dendritic	618	0.01
Megakaryocyte	474	0.01
pDC	156	0.00
Erythrocyte	43	0.00

Supplemental figure 4c

GEM-X 3′

		Seurat						SingleR					
		pbmcsca			pbmc3k			Mona			HPCA		
Cluster	Cell count	Label	Score	% called	Label	Score	% called	Label	Score	% called	Label	Score	% called
0	14052	CD4+ T	1.00	100.00	CD4+ T	0.72	100.00	CD4+ T	0.15	77.33	T	0.24	99.57
1	8654	CD4+ T	0.92	95.86	CD4+ T	0.99	98.20	CD4+ T	0.12	70.29	T	0.20	98.65
2	7168	CD4+ T	0.91	93.96	CD4+ T	0.87	99.29	CD4+ T	0.07	46.00	T	0.12	90.33
3	7078	Classical monocyte	1.00	99.99	Classical monocyte	1.00	99.82	Monocyte	0.05	89.29	Monocyte	0.00	70.91
4	6520	Classical monocyte	1.00	99.57	Classical monocyte	0.94	98.01	Monocyte	0.05	89.46	Monocyte	0.00	71.58
5	5768	NK	0.85	78.59	NK	0.96	92.44	NK	0.00	83.44	NK	0.31	96.27
6	5724	CD4+ T	1.00	100.00	CD4+ T	0.74	100.00	CD4+ T	0.14	75.98	T	0.25	99.74
7	5674	CD8+ T	0.86	97.30	CD8+ T	0.86	93.14	T	0.01	53.03	T	0.11	81.14
8	5566	B	1.00	100.00	B	1.00	100.00	B	0.09	83.27	B	0.07	92.63
9	5477	CD8+ T	0.82	86.12	CD8+ T	0.78	78.46	T	0.01	54.06	T	0.10	56.95
10	5131	CD4+ T	0.83	70.80	CD4+ T	0.93	89.42	T	0.01	60.67	T	0.17	97.19
11	5011	B	1.00	100.00	B	1.00	99.94	B	0.09	87.13	B	0.07	90.46
12	4575	CD4+ T	0.98	99.93	CD4+ T	0.97	100.00	CD4+ T	0.14	79.48	T	0.23	99.23
13	4054	CD8+ T	0.78	86.95	CD8+ T	0.80	86.85	NK	0.00	45.58	T	0.15	93.49
14	3581	CD4+ T	1.00	99.94	CD4+ T	0.66	100.00	T	0.01	75.34	T	0.21	99.47
15	3273	CD4+ T	1.00	100.00	CD4+ T	0.66	100.00	T	0.01	77.05	T	0.20	99.08
16	3262	CD4+ T	0.99	99.91	CD4+ T	0.93	100.00	CD4+ T	0.15	81.85	T	0.24	99.69
17	2955	Non–classical monocyte	0.88	87.01	Non–classical monocyte	1.00	89.75	Monocyte	0.06	87.68	Monocyte	0.00	81.96
18	2808	CD4+ T	0.97	99.93	CD4+ T	0.94	100.00	CD4+ T	0.11	68.13	T	0.19	98.50
19	2434	Megakaryocyte	1.00	68.00	Megakaryocyte	0.97	68.00	B	0.07	45.81	Megakaryocyte	0.07	50.58
20	2081	Classical monocyte	0.93	64.30	Classical monocyte	0.94	26.86	Monocyte	0.02	47.62	Granulocyte	0.10	36.71
21	1962	CD8+ T	0.69	64.53	CD4+ T	0.69	41.54	NK	0.00	55.86	NK	0.16	53.01
22	1821	B	1.00	78.91	B	1.00	73.92	B	0.07	57.11	B	0.06	43.05
23	1600	Dendritic	0.66	59.81	Dendritic	0.96	81.00	Monocyte	0.00	44.44	Monocyte	0.00	58.13
24	501	Classical monocyte	0.48	74.05	CD4+ T	0.69	99.60	B	0.00	47.31	BM	0.01	44.31
25	440	CD4+ T	0.68	72.95	CD4+ T	0.69	79.77	NK	0.00	36.59	T	0.11	81.82
26	370	pDC	0.54	60.54	Dendritic	0.80	75.68	Dendritic	0.10	80.81	Myeloid	0.00	38.38
27	205	Classical monocyte	0.90	93.17	Non–classical monocyte	0.69	45.85	Monocyte	0.06	63.90	Monocyte	0.06	69.27
28	107	Classical monocyte	0.70	68.22	CD4+ T	0.60	74.77	Granulocyte	0.08	88.79	Granulocyte	0.06	51.40
29	62	CD4+ T	0.79	77.42	CD4+ T	0.93	95.16	NK	0.00	32.26	NK	0.11	59.68

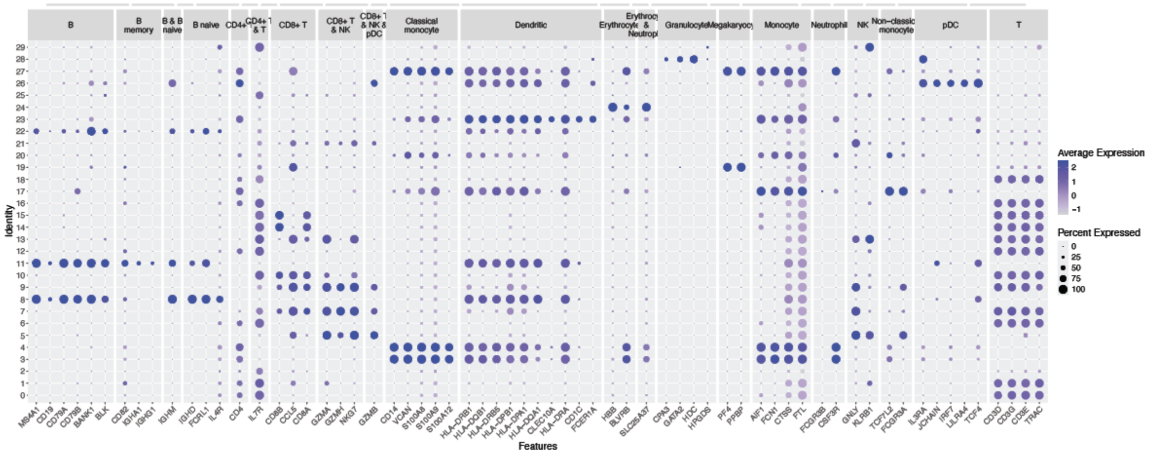

Leiden clustering

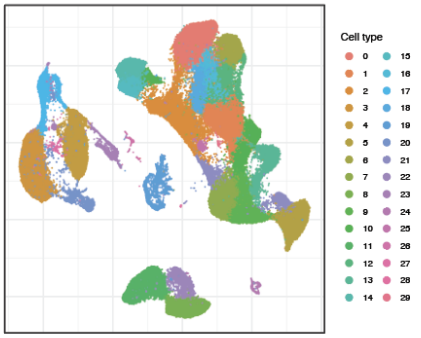

Final annotations

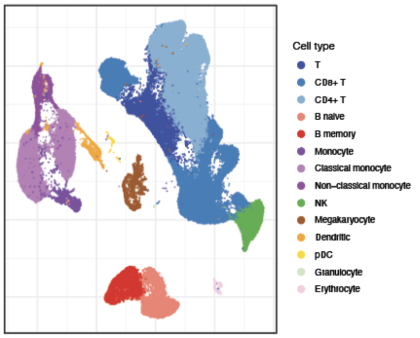

Cell label	Count	Proportion
CD4+ T	39075	0.33
CD8+ T	29152	0.25
Classical monocyte	13803	0.12
T	7670	0.07
B naive	7387	0.06
NK	5768	0.05
B memory	5011	0.04
Non–classical monocyte	2955	0.03
Megakaryocyte	2434	0.02
Monocyte	2081	0.02
Dendritic	1600	0.01
Erythrocyte	501	0.00
pDC	370	0.00
Granulocyte	107	0.00

Supplemental figure 4d

Fluent v4

		Seurat						SingleR					
		pbmcsca			pbmc3k			Mona			HPCA		
Cluster	Cell count	Label	Score	% called	Label	Score	% called	Label	Score	% called	Label	Score	% called
0	4493	NK	0.79	65.57	NK	0.89	72.71	Granulocyte	0.00	27.35	NK	0.11	79.10
1	4311	CD8+ T	0.90	96.10	CD8+ T	0.86	88.66	Granulocyte	0.02	26.68	T	0.08	54.49
2	3918	CD4+ T	0.99	99.72	CD4+ T	0.94	100.00	CD4+ T	0.01	39.77	T	0.10	73.05
3	3481	CD4+ T	0.99	99.57	CD4+ T	0.98	99.97	CD4+ T	0.01	36.68	T	0.09	71.07
4	3007	CD8+ T	0.84	82.21	CD8+ T	0.85	84.84	CD8+ T	0.00	23.11	T	0.08	45.73
5	2995	B	1.00	99.97	B	1.00	99.87	B	0.00	27.88	B	0.06	67.41
6	2884	CD4+ T	0.75	58.95	CD4+ T	0.66	79.02	Granulocyte	0.02	27.98	T	0.09	62.14
7	2810	B	1.00	99.75	B	1.00	99.68	B	0.00	26.37	B	0.07	68.01
8	2776	CD4+ T	0.99	99.14	CD4+ T	0.96	99.96	T	0.01	27.92	T	0.09	68.48
9	2738	CD8+ T	0.73	63.11	CD8+ T	0.72	70.38	Granulocyte	0.04	27.17	T	0.08	56.61
10	2561	CD4+ T	0.84	89.65	CD4+ T	0.73	94.38	Granulocyte	0.02	27.33	T	0.09	65.52
11	2516	Classical monocyte	1.00	98.81	Classical monocyte	0.96	92.13	Monocyte	0.00	32.11	Monocyte	0.01	40.46
12	2447	CD4+ T	0.90	94.97	CD4+ T	0.74	98.86	CD4+ T	0.01	36.13	T	0.09	70.17
13	2269	CD4+ T	0.96	97.93	CD4+ T	0.92	100.00	CD4+ T	0.01	35.04	T	0.08	64.74
14	2267	CD4+ T	0.90	96.12	CD4+ T	0.75	98.68	CD4+ T	0.01	38.20	T	0.10	72.25
15	2198	CD4+ T	0.99	99.68	CD4+ T	0.96	100.00	CD4+ T	0.01	40.67	T	0.09	74.39
16	1999	CD4+ T	0.69	58.93	CD4+ T	0.75	76.14	Granulocyte	0.06	30.67	HSPC	0.01	42.17
17	1741	Non–classical monocyte	0.88	82.37	Non–classical monocyte	1.00	82.65	Monocyte	0.05	37.22	Monocyte	0.00	43.48
18	1445	CD4+ T	0.98	78.75	CD4+ T	0.91	81.59	Lymphocyte progenitor	0.11	40.00	T	0.09	67.82
19	1403	CD4+ T	0.94	96.72	CD4+ T	0.88	99.57	Granulocyte	0.01	30.15	T	0.09	69.71
20	1325	CD8+ T	0.78	78.94	CD8+ T	0.78	79.77	Granulocyte	0.02	24.91	T	0.08	48.75
21	1246	CD4+ T	1.00	99.68	CD4+ T	0.97	100.00	T	0.01	33.39	T	0.09	72.79
22	1236	Classical monocyte	1.00	99.76	Classical monocyte	0.94	97.09	Monocyte	0.00	29.05	Monocyte	0.00	41.26
23	798	Classical monocyte	0.96	85.34	Classical monocyte	0.88	77.44	Lymphocyte progenitor	0.10	29.07	HSPC	0.00	44.61
24	720	Dendritic	0.76	84.44	Dendritic	0.96	80.42	Dendritic	0.00	48.61	Monocyte	0.00	53.06
25	594	CD4+ T	0.97	95.62	CD4+ T	0.92	99.33	Granulocyte	0.05	34.51	T	0.08	60.44
26	292	Megakaryocyte	0.78	39.04	Megakaryocyte	0.85	41.10	Granulocyte	0.07	25.34	T	0.08	32.53
27	213	pDC	0.68	94.84	Dendritic	0.75	78.87	Dendritic	0.08	80.75	Monocyte	0.01	23.94
28	33	CD4+ T	0.97	100.00	CD4+ T	0.94	100.00	Granulocyte	0.01	42.42	T	0.10	45.45
29	27	CD4+ T	0.96	92.59	CD4+ T	0.93	100.00	Granulocyte	0.02	37.04	T	0.10	66.67

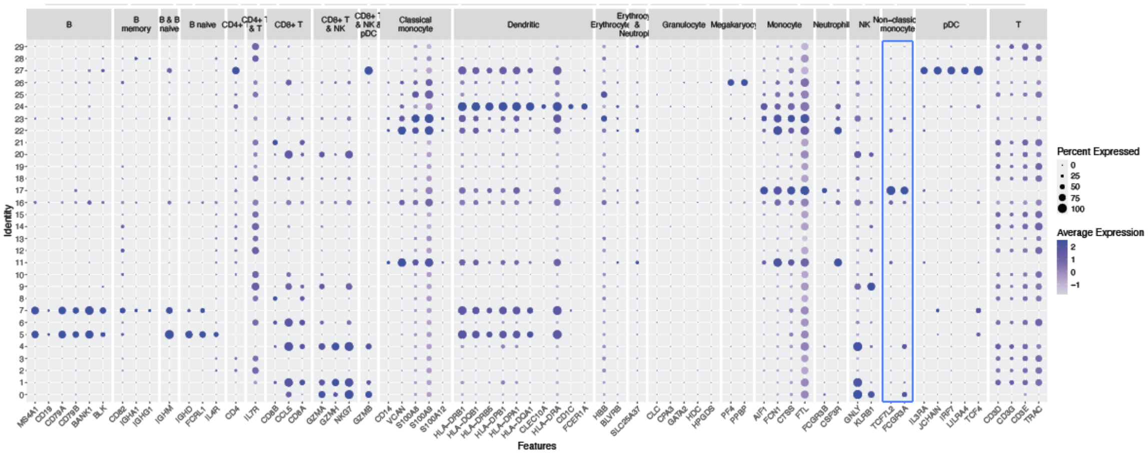

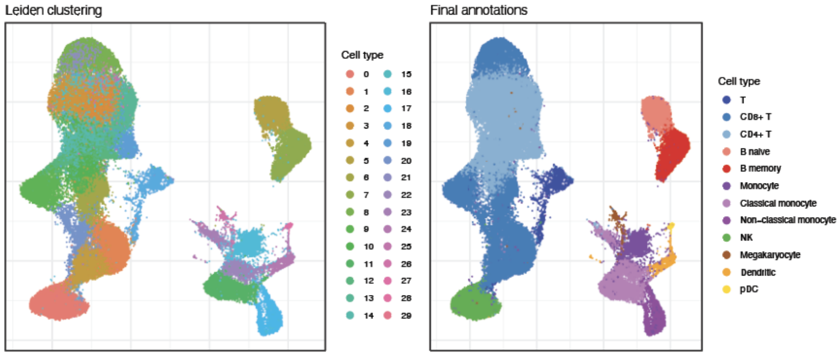

Cell label	Count	Proportion
CD4+ T	21198	0.35
CD8+ T	18287	0.30
Classical monocyte	4550	0.07
NK	4493	0.07
B naive	2995	0.05
B memory	2810	0.05
Monocyte	1999	0.03
Non–classical monocyte	1741	0.03
T	1445	0.02
Dendritic	720	0.01
Megakaryocyte	292	0.00
pDC	213	0.00

Supplemental figure 4e

Fluent V

		Seurat						SingleR					
		pbmcsca			pbmc3k			Mona			HPCA		
Cluster	Cell count	Label	Score	% called	Label	Score	% called	Label	Score	% called	Label	Score	% called
0	4818	CD4+ T	1.00	99.96	CD4+ T	1.00	100.00	CD4+ T	0.01	46.08	T	0.10	81.32
1	4688	CD4+ T	0.96	98.76	CD4+ T	0.88	99.85	CD4+ T	0.06	51.49	T	0.12	90.87
2	4426	NK	0.86	82.94	NK	0.99	91.08	Lymphocyte progenitor	0.06	23.90	NK	0.15	90.99
3	4387	CD8+ T	0.76	51.79	CD4+ T	0.76	53.32	T	0.01	46.61	T	0.10	82.95
4	4177	B	1.00	99.88	B	1.00	99.76	B	0.06	41.18	B	0.06	87.93
5	3597	CD8+ T	0.94	97.22	CD8+ T	0.93	97.22	T	0.01	30.41	T	0.10	77.20
6	3233	CD8+ T	0.86	71.48	CD8+ T	0.85	77.27	T	0.01	33.44	T	0.09	67.34
7	3209	CD4+ T	1.00	99.91	CD4+ T	0.93	100.00	CD4+ T	0.06	46.77	T	0.12	90.06
8	3179	CD4+ T	0.89	86.06	CD4+ T	0.84	82.45	CD4+ T	0.01	41.84	T	0.11	88.83
9	3124	B	1.00	99.90	B	1.00	99.94	B	0.06	51.09	B	0.06	90.69
10	2857	CD4+ T	1.00	99.93	CD4+ T	0.94	100.00	CD4+ T	0.02	48.02	T	0.11	88.20
11	2810	Non–classical monocyte	0.90	85.02	Non–classical monocyte	1.00	87.08	Monocyte	0.06	51.60	Monocyte	0.00	60.00
12	2762	CD4+ T	1.00	99.89	CD4+ T	0.97	100.00	CD4+ T	0.02	46.20	T	0.11	87.55
13	2477	CD4+ T	1.00	99.84	CD4+ T	0.97	99.96	T	0.01	57.77	T	0.10	87.28
14	2458	CD4+ T	0.97	99.63	CD4+ T	0.88	99.92	CD4+ T	0.06	50.28	T	0.12	89.63
15	2416	CD4+ T	1.00	99.96	CD4+ T	0.96	100.00	CD4+ T	0.02	45.45	T	0.12	91.06
16	2404	CD4+ T	1.00	99.83	CD4+ T	0.97	100.00	T	0.01	63.81	T	0.11	90.35
17	2274	CD8+ T	0.74	54.44	CD8+ T	0.92	96.13	NK	0.00	21.68	T	0.10	79.73
18	2027	Classical monocyte	0.99	98.22	Classical monocyte	0.96	98.13	Monocyte	0.00	27.18	Monocyte	0.00	39.42
19	1654	Classical monocyte	1.00	97.88	Classical monocyte	0.87	88.45	Granulocyte	0.10	29.02	Monocyte	0.00	39.18
20	1471	B	1.00	100.00	B	1.00	100.00	B	0.08	52.41	B	0.06	89.19
21	1399	CD4+ T	0.97	94.07	CD4+ T	0.86	95.35	CD4+ T	0.06	38.24	T	0.13	83.85
22	1195	CD4+ T	0.93	96.49	CD4+ T	0.94	99.67	CD4+ T	0.06	46.69	T	0.11	88.45
23	973	Dendritic	0.66	72.76	Dendritic	0.97	86.33	Monocyte	0.00	45.02	Monocyte	0.00	55.19
24	392	pDC	0.59	77.04	Dendritic	0.85	77.30	Dendritic	0.11	80.36	Myeloid	0.00	34.44
25	321	Megakaryocyte	1.00	79.44	Megakaryocyte	0.98	74.14	B	0.23	30.53	Megakaryocyte	0.08	62.93
26	73	Classical monocyte	0.53	98.63	CD4+ T	0.60	98.63	Granulocyte	0.00	36.99	BM	0.00	56.16
27	72	Dendritic	0.60	83.33	Dendritic	0.95	97.22	Dendritic	0.07	72.22	Myeloid	0.00	50.00
28	15	CD4+ T	0.99	86.67	CD4+ T	0.70	100.00	B	0.01	26.67	T	0.09	86.67
29	11	CD4+ T	1.00	100.00	CD4+ T	0.94	100.00	CD4+ T	0.09	36.36	T	0.10	81.82

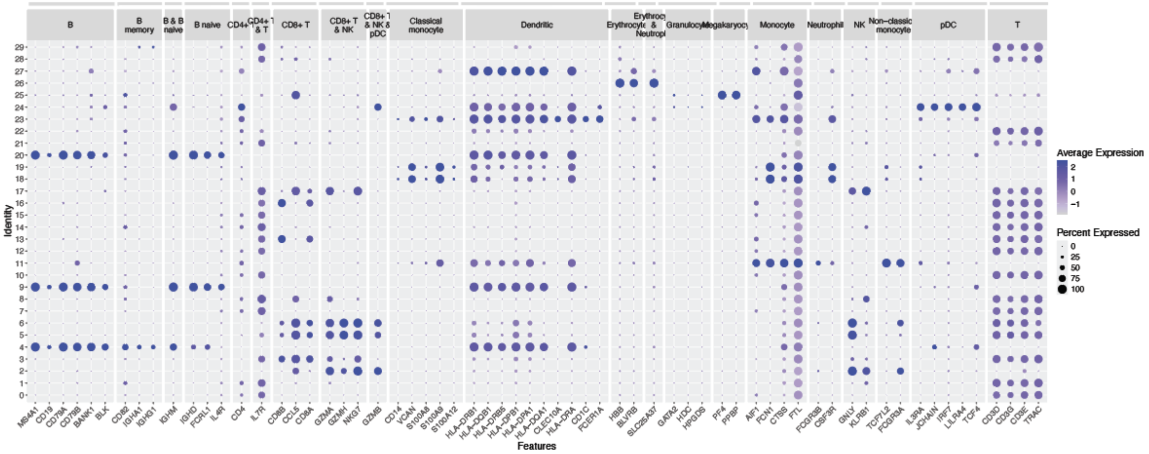

Leiden clustering

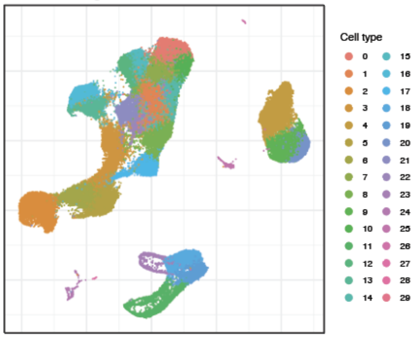

Final annotations

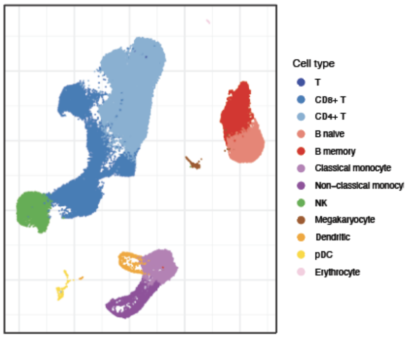

Cell label	Count	Proportion
CD4+ T	28981	0.42
CD8+ T	18387	0.27
B naive	4595	0.07
NK	4426	0.06
B memory	4177	0.06
Classical monocyte	3681	0.05
Non–classical monocyte	2810	0.04
Dendritic	1045	0.02
pDC	392	0.01
Megakaryocyte	321	0.00
Erythrocyte	73	0.00
T	11	0.00

Supplemental figure 4f

Parse v3

		Seurat						SingleR					
		pbmcsca			pbmc3k			Mona			HPCA		
Cluster	Cell count	Label	Score	% called	Label	Score	% called	Label	Score	% called	Label	Score	% called
0	5059	CD8+ T	0.65	49.16	NK	0.73	63.04	NK	0.00	68.16	NK	0.33	94.70
1	4794	CD4+ T	0.99	99.87	CD4+ T	0.78	100.00	CD4+ T	0.07	51.44	T	0.28	96.60
2	4541	CD4+ T	0.96	98.22	CD4+ T	0.75	100.00	CD4+ T	0.08	62.54	T	0.26	95.49
3	4007	CD8+ T	0.73	87.87	CD8+ T	0.69	65.96	NK	0.00	56.48	T	0.10	52.98
4	3932	B	1.00	100.00	B	1.00	99.97	B	0.08	83.21	B	0.12	78.15
5	3910	CD4+ T	0.99	99.90	CD4+ T	0.71	100.00	CD4+ T	0.08	64.02	T	0.27	96.50
6	3865	Classical monocyte	1.00	100.00	Classical monocyte	0.82	95.39	Monocyte	0.02	54.54	Granulocyte	0.13	63.78
7	3863	CD4+ T	0.96	99.77	CD4+ T	0.87	100.00	CD4+ T	0.06	52.78	T	0.18	95.29
8	3766	B	1.00	99.89	B	1.00	99.71	B	0.08	80.72	B	0.13	73.31
9	3667	CD8+ T	0.76	87.13	CD8+ T	0.66	69.40	NK	0.00	75.24	NK	0.21	72.46
10	3071	Classical monocyte	1.00	98.73	Classical monocyte	0.75	80.56	Monocyte	0.02	61.77	Granulocyte	0.13	46.34
11	2885	CD4+ T	1.00	99.83	CD4+ T	0.74	100.00	NK	0.00	40.31	T	0.27	93.97
12	2808	CD4+ T	1.00	100.00	CD4+ T	0.71	100.00	CD4+ T	0.07	55.63	T	0.27	97.01
13	2799	CD4+ T	0.99	99.86	CD4+ T	0.78	100.00	NK	0.00	38.01	T	0.27	95.89
14	2706	Classical monocyte	1.00	99.93	Classical monocyte	0.87	94.12	Monocyte	0.00	45.90	Granulocyte	0.13	65.00
15	2668	Non–classical monocyte	0.71	84.07	Non–classical monocyte	0.98	95.61	Monocyte	0.06	61.77	Granulocyte	0.10	45.88
16	2635	CD4+ T	0.94	97.91	CD4+ T	0.78	99.58	CD4+ T	0.07	58.14	T	0.17	91.65
17	2590	CD4+ T	0.71	68.57	CD4+ T	0.59	62.20	NK	0.00	47.10	T	0.13	72.86
18	2491	CD4+ T	0.86	95.78	CD4+ T	0.76	99.12	CD4+ T	0.06	48.78	T	0.17	88.72
19	2454	CD4+ T	0.73	77.34	CD4+ T	0.73	89.12	NK	0.00	45.60	T	0.14	79.01
20	2137	CD4+ T	0.87	93.12	CD4+ T	0.74	98.46	CD4+ T	0.07	56.76	T	0.16	89.75
21	2136	Classical monocyte	0.94	98.08	Classical monocyte	0.70	75.84	Monocyte	0.05	66.25	Monocyte	0.06	50.51
22	1990	CD4+ T	0.93	98.49	CD4+ T	0.76	99.65	CD4+ T	0.06	51.66	T	0.17	92.46
23	1773	CD4+ T	0.86	97.69	CD4+ T	0.74	99.77	NK	0.00	43.71	T	0.16	86.35
24	818	Classical monocyte	0.52	77.87	Dendritic	0.86	95.11	Monocyte	0.00	41.93	Monocyte	0.07	53.79
25	805	CD4+ T	0.92	97.52	CD4+ T	0.72	99.75	NK	0.00	43.60	T	0.16	83.60
26	615	CD4+ T	0.68	55.77	CD8+ T	0.64	51.38	NK	0.00	69.11	T	0.12	50.89
27	326	pDC	0.69	66.87	Dendritic	0.86	70.55	Dendritic	0.09	65.95	NK	0.01	35.58
28	102	CD4+ T	0.72	82.35	CD4+ T	0.71	79.41	B	0.12	48.04	T	0.12	60.78
29	68	Classical monocyte	0.46	80.88	Dendritic	0.81	95.59	Dendritic	0.07	61.76	NK	0.01	30.88

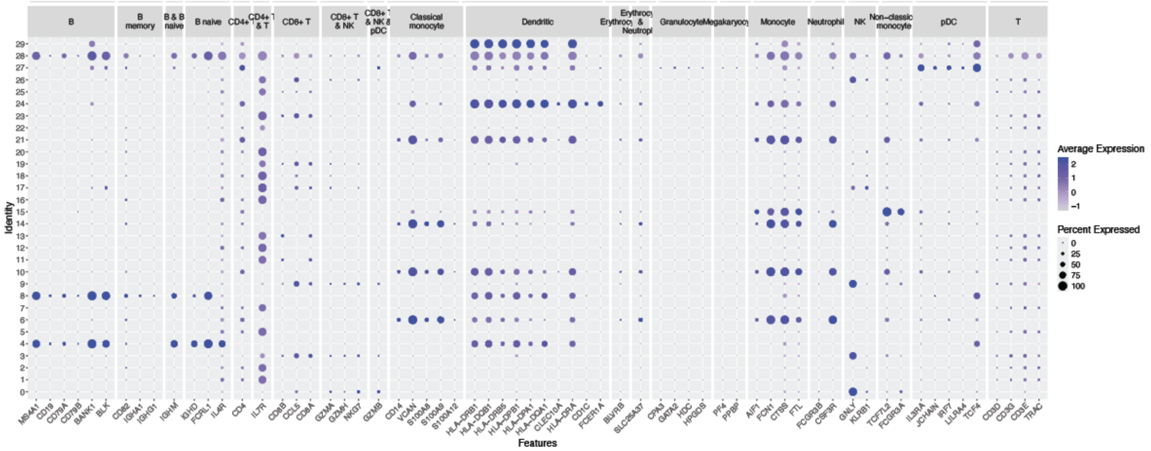

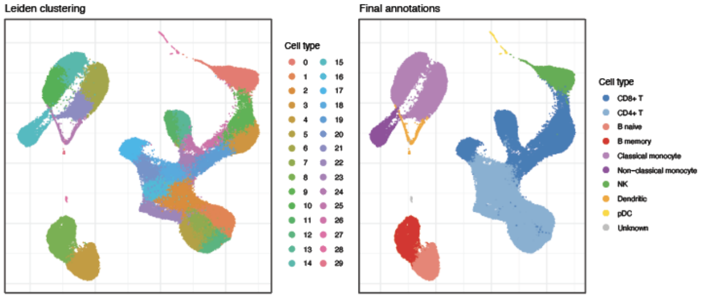

Cell label	Count	Proportion
CD4+ T	29169	0.37
CD8+ T	21595	0.27
Classical monocyte	11778	0.15
NK	5059	0.06
B naive	3932	0.05
B memory	3766	0.05
Non–classical monocyte	2668	0.03
Dendritic	886	0.01
pDC	326	0.00
Unknown	102	0.00

Supplemental figure 4g

Scale

		Seurat						SingleR					
		pbmcsca			pbmc3k			Mona			HPCA		
Cluster	Cell count	Label	Score	% called	Label	Score	% called	Label	Score	% called	Label	Score	% called
0	7538	CD4+ T	1.00	99.83	CD4+ T	0.73	100.00	CD4+ T	0.13	64.57	T	0.17	97.61
1	6923	Classical monocyte	1.00	98.27	Classical monocyte	0.87	88.75	Monocyte	0.00	29.89	Granulocyte	0.13	56.48
2	6626	Classical monocyte	1.00	99.98	Classical monocyte	0.89	98.93	Monocyte	0.00	31.39	Granulocyte	0.13	69.71
3	6546	CD8+ T	0.65	51.74	NK	0.77	69.66	NK	0.00	39.99	NK	0.22	93.43
4	6438	B	1.00	100.00	B	1.00	99.98	B	0.07	76.11	B	0.09	81.30
5	6185	B	1.00	99.95	B	1.00	99.92	B	0.07	68.21	B	0.11	80.02
6	6045	CD4+ T	0.99	99.88	CD4+ T	0.86	99.98	CD4+ T	0.11	61.99	T	0.12	95.15
7	5541	CD8+ T	0.79	91.12	CD8+ T	0.73	77.22	NK	0.00	34.69	T	0.09	53.13
8	5142	CD4+ T	0.96	98.17	CD4+ T	0.80	99.73	CD4+ T	0.10	60.81	T	0.14	92.34
9	4472	CD4+ T	1.00	99.96	CD4+ T	0.71	100.00	CD8+ T	0.06	61.23	T	0.14	96.38
10	4460	Classical monocyte	0.93	96.30	Classical monocyte	0.78	86.70	Monocyte	0.00	34.89	Monocyte	0.06	49.57
11	4420	CD4+ T	1.00	100.00	CD4+ T	0.70	100.00	CD4+ T	0.14	72.35	T	0.16	97.85
12	4394	CD4+ T	0.99	99.41	CD4+ T	0.75	100.00	CD4+ T	0.13	71.37	T	0.18	97.66
13	4282	CD8+ T	0.80	90.99	CD8+ T	0.71	79.10	NK	0.00	47.45	NK	0.11	66.14
14	3836	CD4+ T	0.74	80.27	CD4+ T	0.63	56.60	NK	0.00	27.42	T	0.11	80.27
15	3717	CD4+ T	0.92	97.96	CD4+ T	0.76	99.38	CD4+ T	0.07	50.07	T	0.13	89.86
16	3122	CD4+ T	0.81	77.10	CD4+ T	0.71	81.42	CD8+ T	0.00	30.27	T	0.11	83.79
17	2775	CD4+ T	0.92	98.13	CD4+ T	0.76	99.39	CD8+ T	0.02	48.32	T	0.12	90.85
18	2672	Non–classical monocyte	0.74	89.75	Non–classical monocyte	1.00	93.75	Monocyte	0.01	44.35	Granulocyte	0.10	41.62
19	2662	CD4+ T	1.00	99.89	CD4+ T	0.74	100.00	CD8+ T	0.06	54.51	T	0.13	95.00
20	2657	CD4+ T	0.94	98.08	CD4+ T	0.78	99.55	CD4+ T	0.09	62.85	T	0.13	92.89
21	2571	CD4+ T	0.96	98.95	CD4+ T	0.79	99.65	CD4+ T	0.08	52.94	T	0.11	94.83
22	1723	CD4+ T	0.99	99.59	CD4+ T	0.76	100.00	CD4+ T	0.11	68.49	T	0.14	94.78
23	1415	Classical monocyte	0.52	61.27	Dendritic	0.88	93.99	Dendritic	0.08	35.76	Monocyte	0.05	59.43
24	1060	CD4+ T	0.98	98.11	CD4+ T	0.71	97.36	NK	0.00	26.51	T	0.12	88.30
25	823	Classical monocyte	0.98	77.28	Classical monocyte	0.82	58.93	Monocyte	0.05	27.70	Granulocyte	0.14	48.85
26	621	CD4+ T	0.95	47.34	CD4+ T	0.84	49.76	CD4+ T	0.10	29.31	T	0.11	62.16
27	471	CD8+ T	0.52	42.25	NK	0.62	46.28	NK	0.00	32.27	NK	0.20	80.04
28	358	pDC	0.65	97.49	Dendritic	0.77	95.25	Dendritic	0.09	77.09	Monocyte	0.00	20.11
29	131	CD4+ T	0.99	99.24	CD4+ T	0.73	100.00	CD4+ T	0.08	61.83	T	0.13	96.18

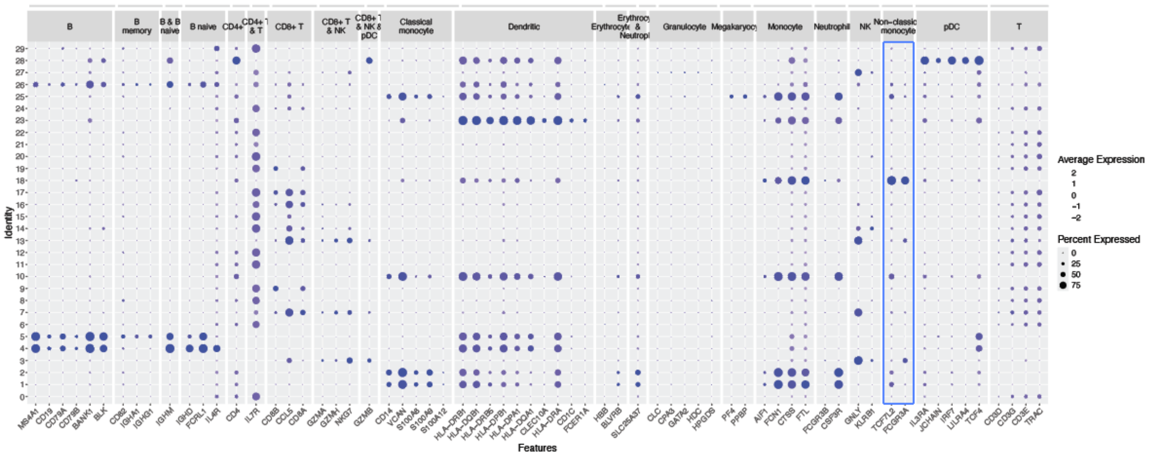

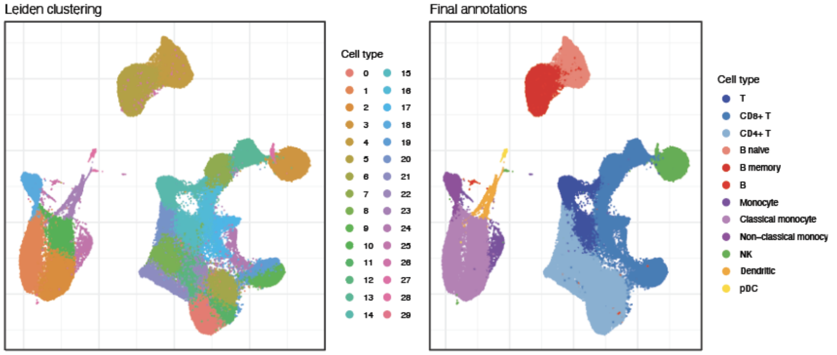

Cell label	Count	Proportion
CD4+ T	34621	0.32
CD8+ T	23914	0.22
Classical monocyte	18009	0.16
T	7553	0.07
NK	7017	0.06
B naive	6438	0.06
B memory	6185	0.06
Non–classical monocyte	2672	0.02
Dendritic	1415	0.01
Monocyte	823	0.01
B	621	0.01
pDC	358	0.00

Supplementary Figure 4: Celltype annotation

Reference mapping data and marker genes used to annotate clusters of cells. Each kit has one set of figures. The first table shows the top annotation call for each reference annotation used. "Cluster" indicates the unsupervised cluster being annotated. The score column indicates the the annotation method's confidence in the annotated label (where closer to 1 is better), averaged over all cells with that label. The "%" column indicates the percentage of cells in that cluster with that label. The dotplot below the table shows the normalized expression of canonical marker genes (x axis) in each cluster (y axis). Bigger dots indicate the gene was detected in more cells, while more saturated color values indicate higher expression. The UMAPs below the dotplot show the 30 unsupervised clusters (left) and their labels (right). The table in the bottom right shows the final count and proportion of each celltype.

Supplemental figure 5a

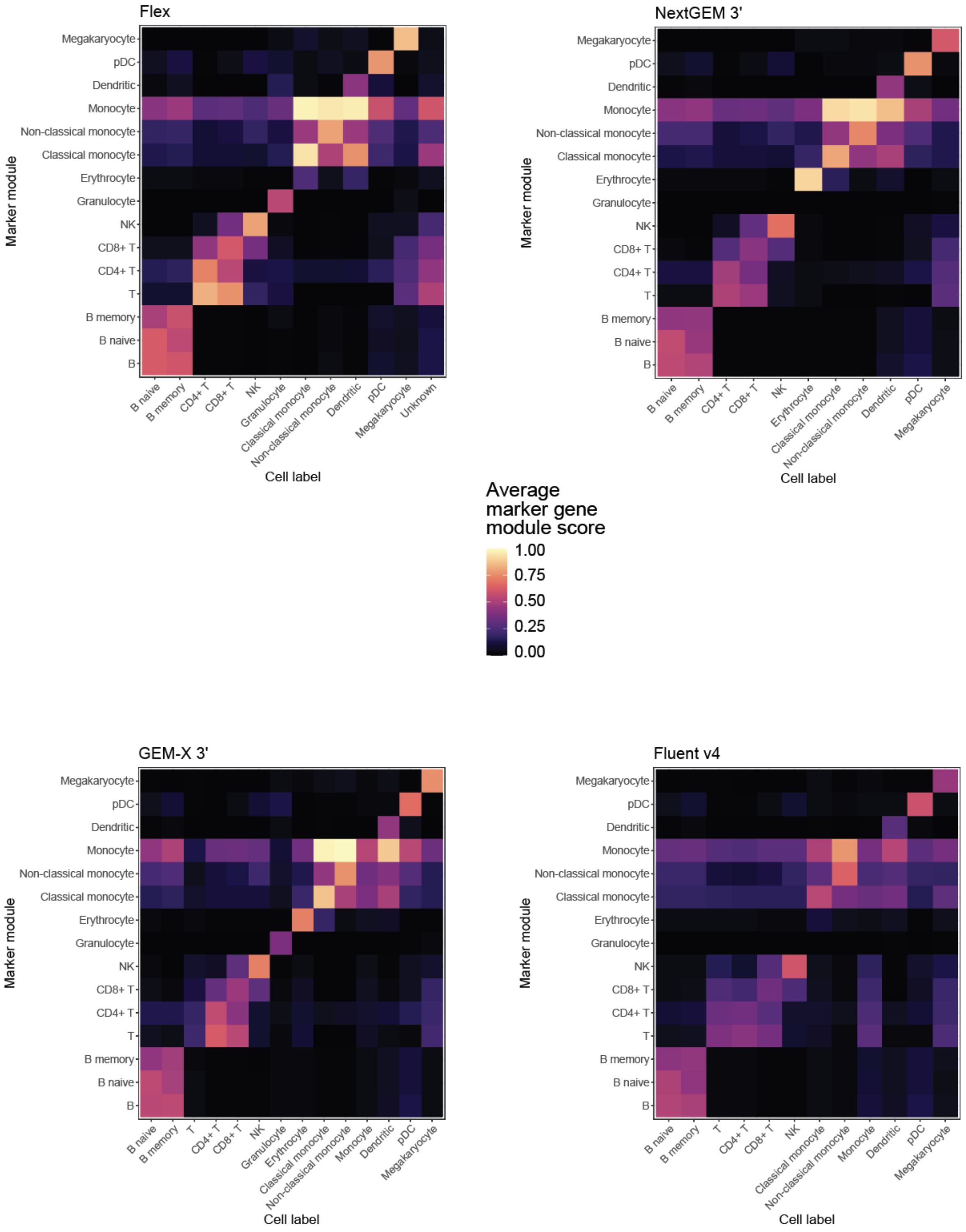

Supplemental figure 5b

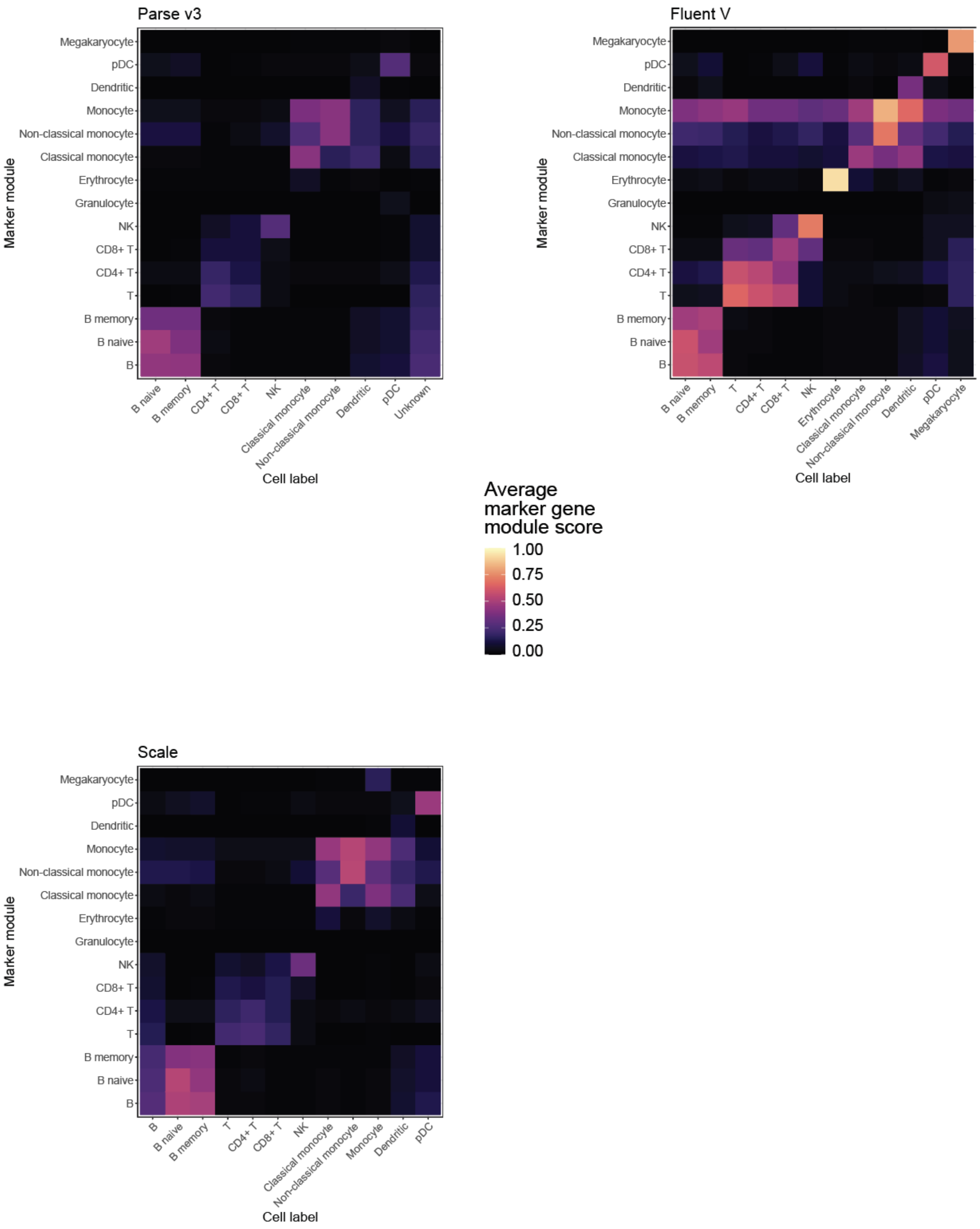

Supplementary Figure 5: Celltype marker module confusion matrices

The expression of celltype marker genes is approximated by a module score, where a higher score indicates those genes are expressed more highly than would be expected by chance. The module score is averaged across cells with the same label within a kit. For example, in the Flex data, the B naive cells (first column) had high expression of “B” and “B naïve” markers, with relatively lower expression of “B memory” and “Monocyte” markers, and little or no expression of “NK” and “megakaryocyte” markers.

Supplemental table 1: All metrics from the study. Where applicable, values were averaged for the kit and reported with a standard error. Some metrics were only calculated at a kit level.

	Metric	Recovered cell #	% recovery (of loaded cells)	High quality cell recovery % (of loaded cells)	% multiplets (of recovered cells)	% Low quality cells (of recovered cells)	Cost per recovered cell	% of reads mapped to transcriptome	% of reads mapped and in called cells	% Ambient RNA
		Cell recovery						Sequencing		
		Figure 2a/b					Figure 2c	Supplemental Figure 2 a-b		Supplemental Figure 2 c-d
	Unit									
	kit level aggregation function	average ± standard error	average ± standard error	average ± standard error	average ± standard error	average ± standard error		average ± standard error	average ± standard error	average ± standard error
Benchmark 1	Flex	10195 ± 324	60.4% ± 1.9%	53% ± 1.4%	10% ± 0.5%	2.3% ± 0.1%	$0.107	94.3% ± 0.1%	88.9% ± 0%	1.2% ± 0.1%
	F1A	10,024	59.4%	52.8%	9.0%	2.1%		94.47%	88.90%	1.20%
	F1B	10,952	64.9%	56.2%	11.2%	2.2%		94.11%	88.95%	1.30%
	F5A	10,397	61.6%	53.5%	10.7%	2.5%		94.27%	88.90%	1.40%
	F5B	9,408	55.8%	49.3%	9.3%	2.3%		94.49%	88.97%	1.00%
	NextGEM	14808 ± 905	62.6% ± 0.3%	51.7% ± 0.4%	10.3% ± 0.1%	7.1% ± 0.3%	$0.230	72.5% ± 0.3%	68.2% ± 0.3%	1.1% ± 0.1%
	F1A	16,338	62.7%	52.1%	10.0%	6.9%		72.18%	68.10%	1.10%
	F1B	16,403	62.9%	51.6%	10.1%	7.9%		72.28%	67.89%	1.30%
	F5A	13,078	61.7%	50.8%	10.6%	7.0%		73.43%	69.05%	1.00%
	F5B	13,413	63.3%	52.5%	10.5%	6.5%		72.14%	67.87%	0.90%
	GEM-X	34761 ± 1763	83.8% ± 1.2%	71.1% ± 1.5%	11.6% ± 0.5%	3.5% ± 0.2%	$0.083	64.7% ± 0.5%	59.8% ± 0.6%	2.4% ± 0%
	F1A	37,147	81.0%	68.1%	12.0%	3.9%		66.12%	61.35%	2.30%
	F1B	38,360	83.6%	69.8%	12.9%	3.7%		63.97%	59.39%	2.50%
	F5A	32,383	86.9%	74.8%	10.7%	3.2%		64.67%	59.54%	2.40%
	F5B	31,155	83.6%	71.9%	10.9%	3.1%		63.86%	58.76%	2.30%
	Fluent v4	16831 ± 787	41.7% ± 1.6%	37.6% ± 2%	8.1% ± 0.5%	1.8% ± 1.5%	$0.060	77.4% ± 0.8%	37.1% ± 0.7%	8.3% ± 0.5%
	F1A	18,953	45.7%	42.3%	7.3%	0.2%		79.63%	37.82%	9.20%
	F1B	16,574	41.4%	38.1%	7.8%	0.3%		77.52%	34.98%	9.00%
	F5A	16,646	41.6%	37.4%	9.6%	0.6%		75.92%	38.24%	7.60%
	F5B	15,151	37.9%	32.6%	7.7%	6.3%		76.63%	37.22%	7.40%
	Fluent V	18331 ± 318	45.9% ± 0.9%	43.1% ± 1%	3.6% ± 0.2%	2.5% ± 0.7%	$0.052	73.2% ± 2.9%	58.5% ± 3.3%	1.8% ± 0.2%
	F1A	18,709	46.5%	44.1%	3.7%	1.4%		66.76%	51.42%	2.10%
	F1B	17,607	43.8%	41.2%	3.9%	2.1%		71.30%	55.18%	1.90%
	F5A	18,010	45.3%	41.6%	3.7%	4.4%		80.48%	66.11%	1.30%
	F5B	18,997	47.8%	45.4%	3.1%	1.9%		74.41%	61.48%	1.70%
	Parse v3	22749 ± 1886	24.7% ± 3%	21.5% ± 2.5%	11.6% ± 0.5%	1.3% ± 0.2%	$0.145	38.1% ± 1.1%	33.8% ± 1%	1.8% ± 0.1%
	F1A	26,679	32.7%	28.1%	12.9%	1.2%		35.84%	31.98%	1.90%
	F1B	18,961	18.9%	16.4%	11.8%	1.7%		37.40%	32.53%	1.80%
	F5A	25,214	25.7%	22.6%	10.8%	1.0%		40.88%	36.63%	1.70%
	F5B	20,142	21.7%	19.1%	10.9%	1.1%		38.31%	34.04%	1.60%
	Scale	29687 ± 5046	23.7% ± 4%	21.9% ± 4.1%	8.8% ± 2.9%	0.2% ± 0.1%	$0.078	63.3% ± 1.4%	57.3% ± 2.7%	1.9% ± 0.3%
	F1A	15,782	12.6%	10.4%	17.4%	0.3%		60.15%	49.94%	2.70%
	F1B	30,025	24.0%	22.4%	6.2%	0.6%		62.70%	57.61%	1.40%
	F5A	33,298	26.6%	25.2%	5.4%	0.0%		66.70%	62.60%	1.40%
	F5B	39,642	31.7%	29.7%	6.3%	0.0%		63.70%	59.19%	1.90%
Benchmark 2	NextGEM 5'	8411 ± 928	48.9% ± 5.6%	42.1% ± 4.3%	9.5% ± 1.9%	4.1% ± 0.8%	$0.279	70.8% ± 0%	68.7% ± 0.2%	0.5% ± 0%
	F1	7,098	41.0%	36.0%	6.9%	5.3%		70.89%	68.47%	0.50%
	F2	9,723	56.8%	48.2%	12.1%	3.0%		70.76%	68.90%	0.50%
	F4									
	F5									
	GEM-X 5'	20137 ± 1174	67.7% ± 4%	58.4% ± 3.8%	8.4% ± 0.3%	5.3% ± 0.8%	$0.099	63.8% ± 1.2%	61.1% ± 1.4%	0.5% ± 0%
	F1	18,103	59.5%	50.5%	8.0%	7.1%		64.52%	61.28%	0.50%
	F2	22,708	75.7%	65.5%	9.2%	4.1%		65.76%	63.74%	0.40%
	F4	19,601	67.8%	59.1%	8.1%	4.8%		61.02%	58.23%	0.50%
	F5	21,211	72.6%	61.3%	9.0%	6.5%		59.73%	56.32%	0.90%
	Parse v2	23005 ± 462	24.8% ± 0.9%	23.3% ± 0.8%	5.3% ± 0.5%	0.7% ± 0.1%	$0.135	53.9% ± 0.6%	48.7% ± 0.7%	1.8% ± 0.1%
	F1	23,921	26.9%	25.0%	6.1%	1.0%		53.10%	48.31%	1.50%
	F2	23,022	23.3%	22.0%	5.6%	0.4%		53.27%	47.57%	2.00%
	F4	22,072	24.2%	23.1%	4.1%	0.7%		55.27%	50.29%	1.80%
	F5	22,285	24.0%	22.9%	3.9%	0.7%		55.74%	51.33%	1.90%

	Metric	Maximum gene estimate	rd50 gene	Maximum UMI estimate	rd50 UMI	Median genes per cell	Median UMI per cell	Median ribosomal read percentage per cell	Median mitochondrial read percentage per cell	Unique gene count	% of reads assigned to 'informative' genes (not ribosomal, mitochondrial, lncRNA, unidentified etc.)	Percentage of reads from unique genes
		Sequencing				Sensitivity						
		Figure 3a/c		Figure 3b/d		Figure 4a				Figure 4b	Figure 4c	
	Unit		Reads per cell		Reads per cell							
	kit level aggregation function					average ± standard error	average ± standard error	average ± standard error	average ± standard error		average ± standard error	
Benchmark 1	Flex	2,993	4,853	6,229	8,971	2397 ± 32	4282 ± 70	0% ± 0%	1% ± 0%	163	95.7% ± 0%	2% ± 0%
	F1A					2342	4165	0.0%	1.0%		95.6%	2.0%
	F1B					2341	4157	0.0%	1.1%		95.6%	2.0%
	F5A					2457	4412	0.0%	1.0%		95.7%	1.9%
	F5B					2449	4395	0.0%	0.9%		95.7%	1.9%
	NextGEM	2,710	7,333	9,032	14,293	2080 ± 32	5628 ± 127	26.9% ± 0.5%	5.9% ± 0.1%	62	81.5% ± 0.1%	4.4% ± 0%
	F1A					2163	5976	27.0%	5.7%		81.6%	4.4%
	F1B					2008	5470	28.2%	5.8%		81.2%	4.4%
	F5A					2085	5655	26.8%	6.2%		81.6%	4.4%
	F5B					2064	5412	25.7%	6.1%		81.6%	4.2%
	GEM-X	3,923	10,895	17,834	32,415	2704 ± 21	7560 ± 92	20.6% ± 0.1%	3.2% ± 0%	693	84.7% ± 0%	3.4% ± 0%
	F1A					2730	7779	20.7%	3.2%		84.7%	3.5%
	F1B					2643	7357	20.8%	3.2%		84.7%	3.4%
	F5A					2731	7630	20.5%	3.3%		84.8%	3.4%
	F5B					2713	7472	20.4%	3.3%		84.8%	3.4%
	Fluent v4	1,479	5,644	3,146	10,233	1159 ± 42	2087 ± 66	22.4% ± 0.9%	4.4% ± 0.1%	90	80.1% ± 0.3%	4.6% ± 0%
	F1A					1188	2139	23.0%	4.2%		80.1%	4.6%
	F1B					1190	2120	22.1%	4.2%		80.4%	4.6%
	F5A					1223	2193	20.2%	4.7%		80.7%	4.6%
	F5B					1036	1894	24.4%	4.4%		79.2%	4.6%
	Fluent V	1,721	5,679	4,882	9,114	1433 ± 43	3616 ± 121	34% ± 0.8%	5.6% ± 0.1%	530	78.3% ± 0.3%	4.7% ± 0.1%
	F1A					1359	3374	34.0%	5.6%		78.1%	4.9%
	F1B					1397	3482	34.4%	5.7%		78.1%	4.8%
	F5A					1418	3681	35.8%	5.4%		77.8%	4.5%
	F5B					1557	3925	31.8%	5.6%		79.2%	4.7%
	Parse v3	2,563	10,732	6,857	24,452	1761 ± 41	3365 ± 136	0.7% ± 0%	4.4% ± 0.2%	2162	90.7% ± 0.1%	4.8% ± 0.1%
	F1A					1753	3306	0.7%	4.2%		90.8%	4.7%
	F1B					1670	3067	0.7%	4.1%		90.8%	4.6%
	F5A					1754	3364	0.6%	5.0%		90.6%	4.8%
	F5B					1868	3724	0.6%	4.4%		90.5%	4.9%
	Scale	2,497	4,798	5,140	9,058	2037 ± 106	3585 ± 321	1% ± 0.1%	0.6% ± 0%	10946	87.4% ± 0.3%	7.1% ± 0.1%
	F1A					1834	3000	1.1%	0.5%		87.1%	7.2%
	F1B					1931	3256	1.3%	0.6%		86.9%	7.2%
	F5A					2324	4469	0.8%	0.7%		88.1%	6.6%
	F5B					2058	3613	0.9%	0.6%		87.3%	7.1%
Benchmark 2	NextGEM 5'	2,213	5,771	7,197	9,647	1784 ± 19	5072 ± 63	35.7% ± 1.4%	3.2% ± 0.2%		81.1% ± 0.3%	0.7% ± 0%
	F1					1810	4982	33.7%	3.5%		81.5%	0.7%
	F2					1757	5161	37.6%	3.0%		80.7%	0.8%
	F4					850	2266	43.7%	1.8%			
	F5					668	1598	43.3%	2.9%			
	GEM-X 5'	3,484	10,471	16,391	26,590	2465 ± 21	8020 ± 42	32.3% ± 0.6%	3.9% ± 0.2%		82.7% ± 0.2%	0.7% ± 0%
	F1					2499	8106	31.1%	4.3%		82.8%	0.7%
	F2					2419	8014	33.4%	4.0%		82.3%	0.8%
	F4					2477	7940	32.5%	3.4%		82.9%	0.8%
	F5					2517	7707	29.9%	4.6%		83.0%	0.9%
	Parse v2	2,483	6,324	6,876	14,396	1965 ± 26	4258 ± 84	1.1% ± 0.1%	3.9% ± 0.2%		90.1% ± 0.1%	2.8% ± 0%
	F1					1953	4167	1.0%	3.6%		90.2%	2.8%
	F2					2022	4452	1.1%	3.8%		90.0%	2.9%
	F4					1920	4156	1.3%	4.4%		90.2%	2.8%
	F5					1876	3930	1.2%	3.6%		89.6%	3.1%

	Metric	Earth mover's distance	Percent of residual variance per gene	Successfully detected enriched pathways	Cost per unique clonotype (1+TRA 1+TRB paired clonotypes only)	Cost per unique clonotype (including partial)	Cost per recovered clone (1+TRA 1+TRB paired clones only)	Cost per recovered clone (including partial)	T cells recovered with clean TRA;TRB clones	Proportion of loaded cells recovered with clean TRA;TRB clones	Inverse Simpson index
		Biological resolution			TCR recovery						
		Figure 6a	Figure 6b	Figure 6c	Figure 2d					Figure 2b	Figure 2f
	Unit	mean ± CI	median (IQR: Q1-Q3)	Number correctly reported of 6 expected							
	kit level aggregation function								average ± standard error	average ± standard error	
Benchmark 1	Flex		9 (3-26) %	4/6							
	F1A	0.06 ± 0.01									
	F1B										
	F5A	0.07 ± 0.01									
	F5B										
	NextGEM		15 (5-38) %	2/6							
	F1A	0.28 ± 0.08									
	F1B										
	F5A	0.14 ± 0.03									
	F5B										
	GEM-X		12 (4-33) %	3/6							
	F1A	0.21 ± 0.03									
	F1B										
	F5A	0.12 ± 0.03									
	F5B										
	Fluent v4		44 (17-81) %	2/6							
	F1A	0.34 ± 0.1									
	F1B										
	F5A	1.38 ± 0.21									
	F5B										
	Fluent V		26 (9-60) %	3/6							
	F1A	0.17 ± 0.03									
	F1B										
	F5A	0.35 ± 0.06									
	F5B										
	Parse v3		24 (8-57) %	1/6							
	F1A	0.29 ± 0.05									
	F1B										
	F5A	0.17 ± 0.04									
	F5B										
	Scale		39 (14-77) %	1/6							
	F1A	0.52 ± 0.1									
	F1B										
	F5A	0.29 ± 0.04									
	F5B										
Benchmark 2	NextGEM 5'				$0.39	$0.32	$0.36	$0.29	5765 ± 860	33.5% ± 5.1%	2760 ± 1215
	F1								4,548	26.28%	1042
	F2								6,981	40.80%	4479
	F4										
	F5										
	GEM-X 5'				$0.14	$0.12	$0.12	$0.10	14941 ± 1514	50.2% ± 5.1%	
	F1								12,313	40.49%	973
	F2								18,251	60.81%	7056
	F4								14,258	49.31%	601
	F5								14,565	49.86%	383
	Parse v2				$0.46	$0.20	$0.40	$0.18	7748 ± 541	8.3% ± 0.4%	
	F1								6,672	7.49%	837
	F2								8,837	8.96%	4884
	F4								7,734	8.49%	471
	F5								7,972	8.60%	624

Supplementary Table 1: Extended metrics

Results for all analyses on each sample with kit-level aggregations and error calculations where appropriate.

Supplemental table 2: Modeled sequencing cost

Projected sequencing costs for each kit normalized for equivalent data recovery. Maximum UMI recovery and RD50 are calculated from a Michaelis-Menten model fit to the observed sequencing saturation data. Reads per cell required to recover a median of 2000 UMI per cell is calculated based on these values. Sequencing price is set at $1.5e-06 per read.

Kit Abbreviation	Projected maximum UMI recovery	rd50	Reads per cell required for 2000 UMI	Normalized sequencing cost (80k cells)	Expected cell recovery	Sequencing cost of whole kit
Flex	6229	8971	4243	$ 509	142956	$ 910
NextGEM3P	9032	14293	4065	$ 488	34154	$ 208
GEMX3P	17834	32415	4094	$ 491	82221	$ 505
Fluent_v4	3146	10233	17865	$ 2,144	60201	$ 1,613
Fluent_V	4882	9114	6325	$ 759	68933	$ 654
Parse_v3	6857	24452	10069	$ 1,208	74281	$ 1,122
Scale	5140	9058	5769	$ 692	109626	$ 949
NextGEM5P	7197	9647	3712	$ 445	28070	$ 156
GEMX5P	16391	26590	3695	$ 443	68486	$ 380
Parse_v2	6876	14396	5905	$ 709	92754	$ 822

Supplementary Table 2: Sequencing cost modeling

Modeling the cost of an example experiment intending to recover the same quantity of transcripts per cell.

Supplemental table 3: Marker genes used for celltype modules and annotation

gene_symbol	expression_le	celltype	relative_to	references
MS4A1	Increased	B	Leukocyte	https://doi.org/10.3390/ijms25073828; https://doi.org/10.3389/fimmu.2023.1223471; https://doi.org/10.1038/s41587-020-0465-8
CD19	Increased	B	Leukocyte	https://doi.org/10.1002/eji.201747168; https://doi.org/10.1038/s41587-020-0465-8; https://doi.org/10.3389/fimmu.2023.1223471
CD79A	Increased	B	Leukocyte	https://doi.org/10.3389/fimmu.2023.1223471; https://doi.org/10.1038/s41587-020-0465-8
CD79B	Increased	B	Leukocyte	https://doi.org/10.3389/fimmu.2023.1223471; https://doi.org/10.1038/s41587-020-0465-8
AFF3	Increased	B	Leukocyte	https://doi.org/10.3389/fimmu.2023.1223471
BANK1	Increased	B	Leukocyte	https://doi.org/10.3389/fimmu.2023.1223471
BLK	Increased	B	Leukocyte	https://doi.org/10.3389/fimmu.2023.1223471
BTLA	Increased	B	Leukocyte	https://doi.org/10.3389/fimmu.2023.1223471
IGHM	Increased	B	Leukocyte	https://doi.org/10.3389/fimmu.2023.1223471
IGHG3	Increased	B	Leukocyte	https://doi.org/10.3389/fimmu.2023.1223471
IL7R	Increased	CD4+ T	Leukocyte	https://doi.org/10.1038/s41587-020-0465-8
CD4	Increased	CD4+ T	Leukocyte	https://doi.org/10.3390/ijms25073828
CD27	Increased	CD4+ T	Leukocyte	https://doi.org/10.1038/s41587-020-0465-8
IL7R	Increased	CD4+ T	CD8+ T	cellxgene
CD8A	Increased	CD8+ T	Leukocyte	https://doi.org/10.3390/ijms25073828
GZMA	Increased	CD8+ T	Leukocyte	https://www.sc-best-practices.org/cellular_structure/annotation.html
GZMB	Increased	CD8+ T	Leukocyte	https://www.sc-best-practices.org/cellular_structure/annotation.html
GZMH	Increased	CD8+ T	Leukocyte	https://www.sc-best-practices.org/cellular_structure/annotation.html
CD8A	Increased	CD8+ T	Leukocyte	https://doi.org/10.1038/s41587-020-0465-8
CD8B	Increased	CD8+ T	Leukocyte	https://doi.org/10.1038/s41587-020-0465-8
CCL5	Increased	CD8+ T	Leukocyte	https://doi.org/10.1038/s41587-020-0465-8
NKG7	Increased	CD8+ T	Leukocyte	https://doi.org/10.1038/s41587-020-0465-8
CD14	Increased	Classical mo	Leukocyte	https://doi.org/10.3390/ijms25073828; https://doi.org/10.1038/s41587-020-0465-8
VCAN	Increased	Classical mo	Leukocyte	https://doi.org/10.1038/s41587-020-0465-8
S100A8	Increased	Classical mo	Leukocyte	https://doi.org/10.1038/s41587-020-0465-8
S100A9	Increased	Classical mo	Leukocyte	https://doi.org/10.1038/s41587-020-0465-8
CSF3R	Increased	Classical mo	Leukocyte	https://doi.org/10.1038/s41587-020-0465-8
LYZ	Increased	Classical mo	Non-classica	https://doi.org/10.1038/s41587-020-0465-8
S100A12	Increased	Classical mo	Leukocyte	https://doi.org/10.1038/s41587-020-0465-8
HLA-DRB5	Increased	Classical mo	Myeloid	cellxgene
FCER1A	Increased	Dendritic	Leukocyte	https://scanpy.readthedocs.io/en/stable/tutorials/basics/clustering-2017.html#finding-marker-genes
CD1C	Increased	Dendritic	Leukocyte	https://doi.org/10.3389/fimmu.2023.1223471
HLA-DRB1	Increased	Dendritic	Leukocyte	https://doi.org/10.3389/fimmu.2023.1223471
HLA-DQB1	Increased	Dendritic	Leukocyte	https://doi.org/10.3389/fimmu.2023.1223471
HLA-DRB5	Increased	Dendritic	Leukocyte	https://doi.org/10.3389/fimmu.2023.1223471

Supp Table 3_marker genes

Ready Accessibility: Good to go Count: 121

Supplementary Table 3: Marker genes

Canonical marker genes used to annotate cells and create celltype module scores.
